# Systems Genetics of Alcoholism

**Published:** 2008

**Authors:** Chantel D. Sloan, Vicki Sayarath, Jason H. Moore

**Keywords:** Alcoholism, alcoholism etiology, genomics, genetics, systems genetics, epistasis, gene–gene interactions, genome-wide studies, biological epistasis, statistical epistasis, risk factors, protective factors, disease etiology, literature review

## Abstract

Alcoholism is a common disease resulting from the complex interaction of genetic, social, and environmental factors. Interest in the high heritability of alcoholism has resulted in many studies of how single genes, as well as an individual’s entire genetic content (i.e., genome) and the proteins expressed by the genome, influence alcoholism risk. The use of large-scale methods to identify and characterize genetic material (i.e., high-throughput technologies) for data gathering and analysis recently has made it possible to investigate the complexity of the genetic architecture of susceptibility to common diseases such as alcoholism on a systems level. Systems genetics is the study of all genetic variations, their interactions with each other (i.e., epistasis), their interactions with the environment (i.e., plastic reaction norms), their relationship with interindividual variation in traits that are influenced by many genes and contribute to disease susceptibility (i.e., intermediate quantitative traits or endophenotypes^1^) defined at different levels of hierarchical biochemical and physiological systems, and their relationship with health and disease. The goal of systems genetics is to provide an understanding of the complex relationship between the genome and disease by investigating intermediate biological processes. After investigating main effects, the first step in a systems genetics approach, as described here, is to search for gene–gene (i.e., epistatic) reactions.

Alcohol addiction is a complex disease that results from a variety of genetic, social, and environmental influences. Alcoholism affected approximately 4.65 percent of the U.S. population in 2001–2002, producing severe economic, social, and medical ramifications (Grant 2004). Researchers estimate that between 50 and 60 percent of alcoholism risk is determined by genetics (Goldman and Bergen 1998; McGue 1999). This strong genetic component has sparked numerous linkage and association studies investigating the roles of chromosomal regions and genetic variants in determining alcoholism susceptibility. To date, some of these studies have identified potential susceptibility genes. However, the complex etiology of alcoholism lends itself to further investigation that takes into account the multiple layers of interaction between genes within the context of both the genome and environment.

Systems genetics offers a new approach to studying the progression of multifaceted diseases such as alcoholism. This new and emerging field is the result of the synergy of disciplines such as bioinformatics, biotechnology, epidemiology, genetics, molecular biology, physiology, psychology, and statistics, all of which contribute to a more complete understanding of the interactions and functions of the entire genome with given ecological and sociological contexts. Detecting, characterizing, and interpreting gene–gene and gene–environment interactions as risk factors for alcoholism is an important first step in a systems genetics approach that combines genomics^2^ and proteomics^3^ data with methods to understand how biological processes work together to determine human health. This approach does not, however, negate the need to look for variants that directly impact disease independent of interaction effects (main effects) within the data.

A complete review of all results from genetic, genomic, proteomic, and metabolic studies of alcoholism is beyond the scope of this review. This article focuses on recent literature involving studies of genes selected based on biochemical evidence for their role in disease (i.e., candidate genes) and genome-wide studies, followed by an overview of the interaction among genes (i.e., epistasis) and its current and potential application in the study of alcoholism. This article concludes with a discussion of several methods currently being developed that incorporate a systems approach to genetics and their potential applications for the future study of alcoholism.

## Alcoholism Genetics: A Brief Overview

The genetic architecture of susceptibility to a disease such as alcoholism can be defined as (1) the number of genes directly or indirectly involved, (2) the interindividual variation in those genes, and (3) the magnitude and nature of their specific genetic effects. Alcoholism develops in susceptible individuals as a result of genetic, environmental (e.g., alcohol consumption), and social influences, as well as their propensity for risk-taking behaviors (Ramoz et al. 2006). Because of this complex etiology, multiple levels of information must be integrated to more completely understand the genetic architecture of alcoholism. In the progression of multifactorial diseases such as alcoholism, gene–gene interactions result in a variety of differentially expressed proteins. These proteins also interact, resulting in certain biochemical and physiological characteristics that, in the presence of certain environmental influences, result in alcoholism. Although studies of alcoholism’s etiology have been successful in identifying a few candidate genes for susceptibility, interindividual variation in these genes accounts for only a small proportion of the overall heritability of the disease. Much of the remaining heritability is potentially due to DNA sequence variations, with effects that are dependent on contexts defined by the rest of the genome and the environment.

This article first reviews what currently is known about the role genetics plays in alcoholism and then gives a brief overview of the key findings from candidate gene and genome-wide studies. These studies confirm the role of genetics in the development of alcoholism and elucidate the need for a systems-based approach to the study of the genetic basis of the disease.

### Candidate Gene Studies

Two basic strategies are used to identify genetic risk factors for common human diseases. The most common approach is to focus on a few candidate genes. This targeted approach is popular because a biological basis exists for the hypotheses being tested. Alternatively, genetic variations (i.e., polymorphisms) from across the human genome can be measured in a high-throughput manner to search for genetic risk factors without making assumptions about which genes might be important. This latter genome-wide approach is popular because much more information is examined. Researchers frequently debate the advantages and disadvantages between candidate gene and genome-wide strategies as well as the differences in genome-wide strategies themselves. Risch (2000) suggests that genome-wide association studies, which compare the genomes of people with an illness (i.e., cases) with unaffected people (i.e., controls), may be more sensitive toward finding effects for complex diseases than genome-wide linkage studies, which seek to identify regions of the genome that are associated with disease risk. The candidate gene approach is more direct and hypothesis-based and, therefore, perhaps more likely to have significant findings, although less likely to find novel associations. The genes most extensively examined by candidate gene studies have been those involved in alcohol (i.e., ethanol) metabolism and in neurological pathways responsible for increased risk taking and “reward” stimulation from ethanol. The metabolic genes most frequently studied include those for the enzymes alcohol dehydrogenase (ADH), aldehyde dehydrogenase (ALDH), catalase, and cytochrome P450 2E1 (CYP2E1). ADH is responsible for 80 percent of ethanol’s metabolism to acetaldehyde, which is then further metabolized to acetate by ALDH. CYP2E1 metabolizes approximately 10 percent of ethanol and, because of its lower affinity for ethanol, is largely active only when ADH is saturated (Gemma et al. 2006).

Researchers also have studied various genes related to the brain chemistry of alcoholism and specific chemicals (i.e., neurotransmitters) involved in addiction. Such research has examined genes for the binding sites (i.e., receptors) for the neurotransmitter gamma-aminobutyric acid (GABA); opioid receptors; components of the pathways for the neurotransmitters serotonin, dopamine, and glutamate, as well as the enzyme catechol-*O*-methyl-transferase (COMT), which is involved in the inactivation of dopamine; and the neurotransmitter neuropeptide Y (NPY) (Dick and Bierut 2006; Oroszi and Goldman 2004).

Candidate gene association studies also help to focus on genetic variants that may be directly linked to pathophysiology, as reviewed by Kohnke (2007). Several genes, including those for neurotransmitters such as dopamine as well as those genes mentioned above, have undergone frequent investigation in candidate association as well as linkage studies. Some have shown promising, and others conflicting, results.

The candidate gene approach has not only confirmed a genetic component of alcoholism but also has brought important understanding to disease etiology and may yield further insight when integrated with gene expression and proteomic analysis. Though the candidate approach has proven useful, genome-wide studies may provide a more comprehensive view of whole-genome interaction in the etiology of alcohol addiction.

### Genome-Wide Studies

A first step toward advancing our understanding of the role of genetics in the development of alcoholism is to gather genetic data on a genome-wide scale. As previously noted, studies to date have focused on a limited number of candidate genes. Although these studies have furthered our understanding of the disease and will continue to play an important role in our understanding of alcoholism, the advent of new genomic and computational methods is making it possible to broaden our knowledge of the disease through a more inclusive whole-genome approach. There are two main approaches to genome-wide analysis—association and linkage. Association studies examine genetic polymorphisms associated with case or control status, whereas linkage studies investigate the inheritance of specific locations on a chromosome (i.e., loci) within family lines. Though these two approaches are quite different in procedure and analysis, both are being greatly advanced by commercially available technology. The discussion of systems genetics below cites examples of both approaches.

The largest genome-wide study of alcoholism to date has been the Collaborative Study on the Genetics of Alcoholism (COGA) (Begleiter et al. 1995; Reich 1996). This study collected data from families with alcoholism and has been used for both linkage and association analyses. Researchers have identified candidate susceptibility regions on chromosomes 1, 2, and 7, including susceptibility and protective regions within the neurexin 1(*NRXN1*) gene on chromosome 2 (Yang et al. 2005). Another study by Namkung and colleagues (2005) was able to show that association analysis of COGA data pointed to a significant gene on chromosome 7, as well as 13 genes associated with both alcoholism and schizophrenia. The genes in these regions represent candidates for independent main effects for susceptibility to alcoholism. Some of the COGA victories include associations that have been subsequently substantiated by other investigations, such as GABA receptor alpha (GABRA2), cholinergic muscarinic 2 receptor (CHRM2), and ADH4 (Edenberg and Froud 2006). The challenge thereafter is to identify DNA sequence variations that influence susceptibility primarily through nonlinear interactions (i.e., the total interaction is not the sum of the influence of its interacting parts) with other genes or environmental factors. The complex interactions resulting in differential disease susceptibility and progression necessitate further investigation of epistatic interactions, which occur when the action of one gene is modified by one or several other genes.

## Epistasis or Gene–Gene Interaction

Epistasis has been defined in multiple ways (e.g., Brodie 2000; Hollander 1955; Phillips 1998). The following section reviews two types of epistasis—biological and statistical (Moore and Williams 2005)—including aspects of biological epistasis, current methods used to study statistical epistasis, and analytical challenges associated with studying epistasis for genome-wide data and possible strategies for overcoming these challenges.

**Figure 1:**
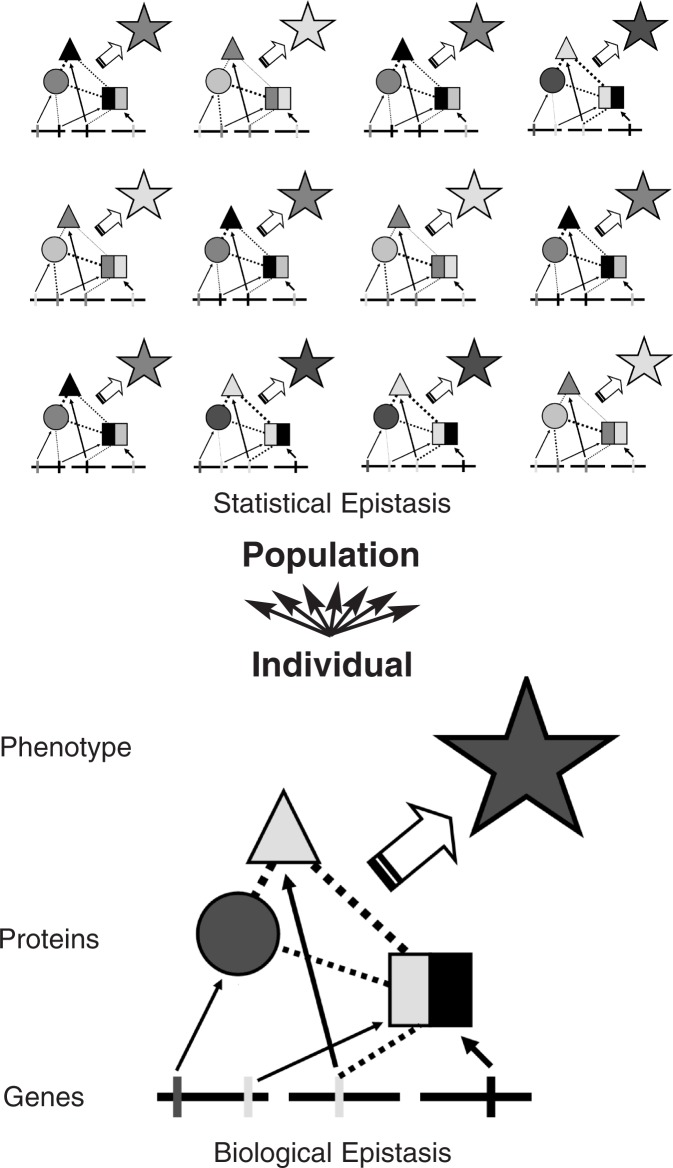
Biological epistasis is a measure of gene interaction occurring within a single organism, via gene–gene, gene–protein, and protein–protein interaction. Statistical epistasis is a detectable measure of epistasis at the population level.

Biological epistasis results from physical interactions among biomolecules (e.g., DNA, RNA, proteins, enzymes, etc.) and occurs at the cellular level in an individual. This type of epistasis is what Bateson (1909) had in mind when he coined the term. Statistical epistasis was first defined by Fisher (1918) as a mathematical phenomenon that occurs at the population level and is realized when there is interindividual variation in DNA sequences. Figure 1 illustrates the conceptual divide between biological and statistical epistasis that is important to understand in order to make biological inferences from statistical results (Moore and Williams 2005).

Understanding biological epistasis is one important motivation for studying statistical epistasis. With alcoholism, researchers have focused more on the direct study of biological epistasis at the cellular and biochemical level. However, a wide range of analytical tools is available for the study of statistical epistasis in human populations that could be applied to this disease. Methods for detecting statistical epistasis, described below, include linear and logistic regression (e.g., Cordell 2002; Millstein et al. 2006), combinatorial partitioning (Nelson et al. 2001), restricted partitioning (Culverhouse et al. 2004), set association analysis (Hoh et al. 2001; Hoh and Ott 2003, 2004; Ott and Hoh 2003; Wille et al. 2003), genetic programming of neural networks (Motsinger et al. 2006; Ritchie 2003*b*;Ritchie et al. 2004; White et al. 2003), symbolic discriminant analysis (Moore et al. 2002, 2007), and multifactor dimensionality reduction (MDR) (Hahn and Moore 2004; Moore 2004, 2007; Moore et al. 2006; Ritchie et al. 2001, 2003*b*). The following section focuses first on studies of biological epistasis and then reviews some of these statistical methods.

### Biological Epistasis and Alcoholism

Though gene–gene interactions are expected to play an important role in alcoholism, few studies have investigated epistasis in this disease. As mentioned above, members of the ADH gene family are common candidates for alcoholism susceptibility genes. As a model system, fruit flies (i.e., *Drosophila*) have been used to study epistasis in ADH genes and genes for other metabolic enzymes in relation to larval tolerance of ethanol. In one study, Freriksen and colleagues (1994) discerned differences in the metabolism of ethanol by measuring the ratios of metabolic intermediates that were “fluxed” through different branches of the ethanol metabolism pathways. They found that ethanol metabolism varied depending on the particular ADH genes present (i.e., ADH genotype). The authors suggested that the changes in intermediate ratios through the pathway might be the foundation for observed statistical epistatic interactions.

The biological epistasis of alcoholism also has been studied in reference to neurological genes. As demonstrated by Job and colleagues (2007), in a study of a type of ethanol-stimulated opioid receptor (i.e., the μ-opioid receptor [MOPr]) in mice, epistatic interactions may be sexually dimorphic. The researchers found a sex–genotype interaction regarding the level of dopamine released in mice with the MOPr gene deleted (i.e., MOPr knockout mice) when they were stimulated with ethanol in the ventral striatum, with females showing a larger reduction.

Palmer and colleagues (2003) found a potential interaction between polymorphisms of the dopamine receptor D2 (*DRD2*) gene by constructing *DRD2* knockout mice against two different genetic backgrounds (B6 and 129). B6 mice previously were shown to have less stimulation in response to ethanol than 129 background mice. The two types of *DRD2* knockouts showed different locomoter stimulator and locomotor sensitization, demonstrating that there was an epistatic interaction between *DRD2* and the genetic background. *DRD2* also has been a popular target for studies of statistical epistasis, as described below.

### Statistical Epistasis and Alcoholism

In contrast to biological epistasis, statistical epistasis is a population-level phenomenon that arises from linear and nonlinear patterns of variation in genotypes and complex traits such as alcoholism. As such, detecting and characterizing statistical epistasis requires special analytical modeling methods. An association study by Osier and colleagues (2004) found a potential epistatic interaction between the ADH1B and ADH7 genes among a Han Chinese population. The ADH variant *ADH1B Arg47His* previously was found to be protective against alcoholism (Osier 1999). This protective effect was not solely related to the *ADH1B* gene but to an interaction with *ADH7* (or a locus that occurs with it more often than would be expected by random chance [i.e., a site in linkage disequilibrium with it]). The study included analysis of sets of closely linked genetic variants that tend to be inherited together (i.e., haplotypes) and 2-x-2 contingency tables, which are used to record and analyze the relationship between two or more variables, to discern a statistically significant, though relatively weak, protective effect of the *ADH7 Sty*I site.

Neurological statistical epistasis studies include a study among three different Taiwanese populations that examined three different *DRD2* polymorphisms. The results showed no association between the *DRD2* polymorphisms and alcoholism when considered individually or as haplotypes (Lu et al. 1996). The minor (A1) allele of *DRD2* and major (G1) allele of GABA receptor beta 3 (GABRB3), however, have been associated with alcoholism risk independently and in combination in a study of severely alcoholic and nonalcoholic Caucasians (Noble et al. 1998). This discrepancy regarding the statistical effect of *DRD2* variations could be due to several factors, including ethnicity or an effect of *DRD2* variation that is only detectable when epistasis is considered. Also, in a recent association study, COGA researchers have found that alcoholism association with the *DRD2* region actually may be the result of an association with the nearby ankyrin repeat and kinase domain containing 1 (ANKK1) gene (Dick et al. 2007).

### Methods for Detecting Statistical Epistasis

Two commonly used statistical methods for studying epistasis are parametric logistic regression and nonparametric MDR. In logistic regression models, the probability of disease (p) is expressed as a linear function of independent variables (see Hosmer and Lemeshow 2002; Kleinbaum and Klein 2002). The advantage of logistic regression is that interactions can be modeled relatively easily, the statistical theory is very well characterized, and the approach can be implemented on a standard desktop computer using a variety of freely and commercially available statistical packages. An important disadvantage is that very large sample sizes are needed to accurately estimate the parameters in the model when there are many independent variables.^4^ Epistasis is difficult to detect and characterize using traditional parametric statistical methods such as logistic regression because of the sparseness of the data in high dimensions. That is, when interactions among multiple polymorphisms are considered, there are many multilocus genotype combinations that have very few or no data points. For example, with two single nucleotide polymorphisms^5^ (SNPs) that each have three genotypes, there are nine two-locus genotype combinations. In the case of three SNPs, there are 27 three-locus genotype combinations. Therefore, as each additional SNP is considered, the number of multilocus genotype combinations increases exponentially. The result of this added dimensionality is that exponentially larger sample sizes are needed in order to have enough data to estimate the interaction effects. This phenomenon has been referred to as the curse of dimensionality (Bellman 1961) and, for methods such as logistic regression, can lead to parameter estimates that have very large standard errors,^6^ resulting in an increase in false-positives (i.e., type I errors) (Concato et al. 1993; Hosmer and Lemeshow 2002; Peduzzi et al. 1996). In addition, detecting gene–gene interactions using traditional procedures for fitting regression models can be problematic, leading to an increase in false negatives (i.e., type II errors) and a decrease in power.

MDR is a data-mining strategy for identifying combinations of SNPs that are predictive of a discrete clinical end point in which no parameters are estimated (i.e., nonparametric) and no genetic model is assumed (i.e., genetic model–free) (Hahn et al. 2003; Hahn and Moore 2004; Moore 2004, 2007; Moore et al. 2006; Ritchie et al. 2001, 2003*b*). At the heart of the MDR approach is a feature or attribute construction algorithm that creates a new attribute (characteristic) by pooling genotypes from multiple SNPs. The process of defining a new attribute as a function of two or more other attributes is referred to as constructive induction or attribute construction and was first developed by Michalski (1983).

**Figure 2:**
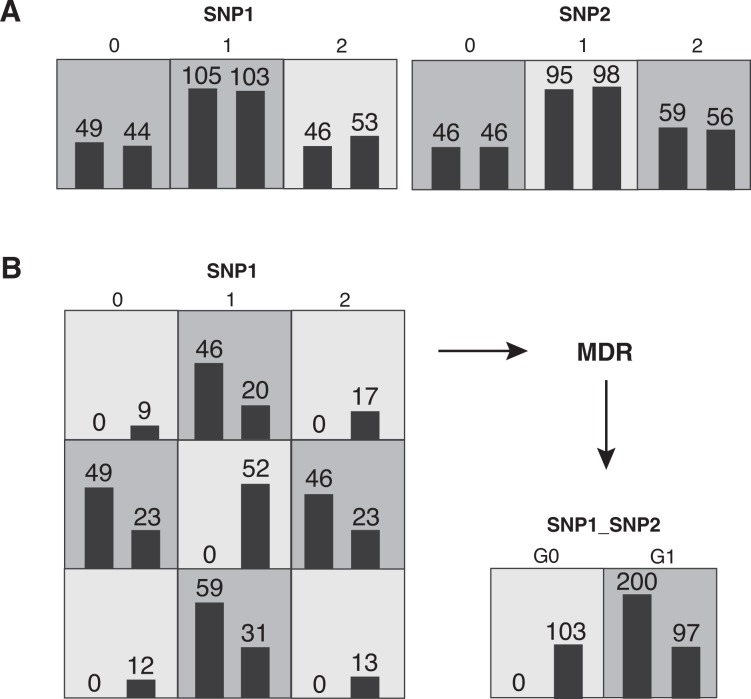
Multifactor dimensionality reduction (MDR) attribute construction. **A)** Distribution of cases (left bars) and controls (right bars) for each of the three genotypes of single nucleotide polymorphism (SNP) 1 and SNP2. The dark-shaded cells have been labeled “high risk,” and the light-shaded cells have been labeled “low risk.” **B)** Distribution of cases and controls when the two functional SNPs are considered jointly. A new single attribute is constructed by pooling the high-risk genotype combinations into one group (G1) and the low-risk genotype into another group (G0).

Constructive induction using MDR is accomplished in the following way. Given a threshold *T,* a multilocus genotype combination is considered high risk if the ratio of cases (subjects with disease) to controls (healthy subjects) exceeds *T;* otherwise, it is considered low risk. Genotype combinations considered to be high risk are labeled G_1_, whereas those considered low risk are labeled G_0_. This process constructs a new one-dimensional attribute with levels G_0_ and G_1_. It is this new single variable that is assessed using a classification method such as naïve Bayes^7^ or logistic regression. Figure 2 illustrates the constructive induction process used in MDR for two interacting SNPs.

The MDR method is based on the idea that reducing the dimensionality of the data will make the detection of attribute dependencies (e.g., the SNP interactions that determine the classification of case/control) easier for a classifier such as a decision tree or a naïve Bayes learner. The drawbacks to this method include computational time for large datasets or interactions beyond four-way. Also, MDR software currently cannot be applied to continuous end points (such as blood pressure) but is very powerful for discrete end points such as “case” or “control” classifications even with missing data and genotyping error (Ritchie et al. 2003*b*). For family-based studies, a version of the software known as the MDR–PDT is available that is based on a merging of MDR and the Pedigree Disequilibrium Test, which measures the transmission of disease alleles through pedigrees. This method has excellent power for detecting epistasis in studies of nuclear families with low phenocopy errors^8^ (Martin et al. 2006). MDR open-source software is freely available from www.epistasis.org.

The MDR method has been successfully applied to the detection of epistasis for a variety of common human diseases, including sporadic breast cancer (Ritchie et al. 2001), essential hypertension (Moore and Williams 2002; Williams et al. 2004), atrial fibrillation (Moore et al. 2006; Tsai et al. 2004), myocardial infarction (Coffey et al. 2004), type 2 diabetes (Cho et al. 2004), prostate cancer (Xu 2005), bladder cancer (Andrew et al. 2006), schizophrenia (Qin et al. 2005; Vilella 2007), autism (Coutinho et al. 2007), and familial amyloid polyneuropathy (Soares et al. 2005). The MDR method also has been successfully applied in the context of studying genetic variation that influences an individual’s response to drugs (i.e., pharmacogenetics) and toxic substances (i.e., toxicogenetics) (e.g., Wilke et al. 2005), and also is likely to be a useful tool for detecting epistasis in genetic studies of alcoholism.

### Genome-Wide Analysis of Epistasis

Technologies now are available to measure 1 million or more SNPs across the human genome. The availability of high-dimensional SNP data has opened the door to genome-wide association studies. The COGA studies have made good progress in using large-scale genotyping studies for alcoholism. As genome-wide methods improve, more studies implementing systems genetics methods are likely to be undertaken.

A number of significant analytical challenges are associated with genome-wide data, as summarized by Hirschhorn and Daly (2005) and Wang and colleagues (2005). Moore and Ritchie (2004) have outlined three significant challenges that must be overcome if we are to successfully identify gene–gene interactions using a genome-wide approach. First, powerful data mining and machine learning methods such as MDR will need to be developed to statistically model the relationship between combinations of DNA sequence variations and disease susceptibility. A second challenge is the selection of genetic features or attributes that should be included for analysis. If interactions between genes explain most of the heritability of common diseases, then combinations of DNA sequence variations will need to be evaluated from a list of thousands of candidates. Methods for doing this are discussed below. The third challenge is the interpretation of gene–gene interaction models. Although a statistical model can be used to identify DNA sequence variations that confer risk for disease, this approach cannot be translated into specific prevention and treatment strategies without interpreting the results in the context of human biology. Making etiological inferences from computational models may therefore be the most important and the most difficult challenge of all (Moore and Williams 2005).

A recent report from the International HapMap Consortium (Altshuler et al. 2005) suggests that approximately 300,000 carefully selected SNPs may be necessary to capture all of the relevant variation across the Caucasian human genome. Assuming this is true, we would need to scan 4.5 × 10^10^ pairwise combinations of SNPs to find what Goldberg (2002) refers to as a genetic needle in a haystack. The number of higher-order combinations is astronomical, which raises the question, “What is the optimal approach to this problem?”

There are two general approaches to selecting attributes for predictive models: the wrapper approach and the filter approach. With a filter approach, researchers analyze a preselected subset of SNPs that most likely are to be significant. With a wrapper approach, researchers use some measure while running the analysis itself to select a subset of SNPs. The key difference between the two approaches is that the classifier (i.e., statistical model) plays no role in selecting which attributes to consider in the filter approach. As Freitas (2002) reviews, the advantage of the filter is speed, whereas the wrapper approach has the potential to do a better job classifying. For examples of each, see Moore and White (2006, 2007).

Though attempts at genome-wide studies of alcoholism have not employed these methods to date, such strategies will be essential in the future to understanding the systems genetics of alcoholism. As genome-wide datasets become available, tools such as MDR for modeling interactions must be developed in conjunction with powerful computational algorithms for searching for optimal combinations of polymorphisms. To understand the link between genotype and an alcoholic phenotype, we must traverse the divide between genes and alcoholism by observing and measuring the levels of gene expression as well as proteomic and environmental interactions that compose the interactive genome.

## Toward a Systems Genetics Approach to the Study of Alcoholism

**Figure 3:**
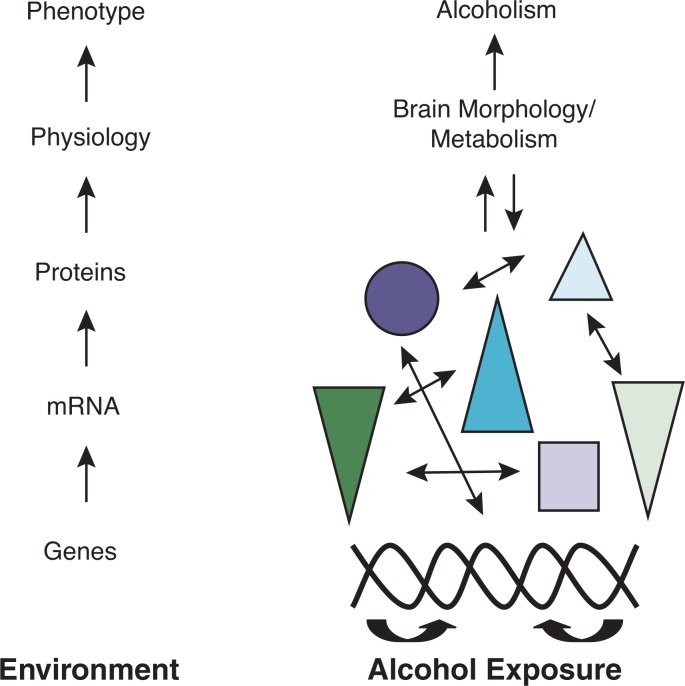
The contribution of genes to alcoholism progresses through a hierarchy of gene expression, protein interaction, and physiology within the context of environment. Though association, linkage, expression, proteomic, physiological, and environmental studies capture pertinent information from each hierarchical level, they do not independently capture the complex interaction actually responsible for disease. Colored shapes represent interacting gene products (i.e., RNA and proteins).

Documenting the role of epistasis in alcoholism as described above is a good first step toward thinking about how genes work together to influence risk. However, a more complete understanding of the hierarchical mapping relationship between genotype and phenotype will come from studying other key biomolecules, such as mRNA and proteins, and their interactions. The ultimate goal of systems genetics is to determine how these biomolecular interactions in a particular ecological context influence the physiological processes that are responsible for disease phenotypes (figure 3). This information will dramatically increase our ability to develop effective prevention and treatment strategies. The studies and strategies reviewed below provide a basis for moving forward with a systems genetics approach to alcoholism.

### Genomics and Proteomics

Microarray analysis, which allows the simultaneous analysis of up to tens of thousands of genes, now is a routine approach for investigating changes in gene expression on a genome-wide scale and has yielded interesting results in regards to alcoholism. Meta-analyses of microarray studies have revealed patterns in gene regulation in alcoholics according to brain region. For example, in chronic alcoholics, DNA binding and cell signaling genes show increased expression in the prefrontal cortex relative to the nucleus accumbens, and genes controlling cellular plasticity were notably downregulated in the nucleus accumbens (Flatscher-Bader et al. 2006; Mulligan et al. 2006; Worst and Vrana 2005). These are examples of studies that combined behavioral genetics and microarray analyses, which can be a promising way to uncover associations between gene expression and behavior patterns (Letwin et al. 2006).

High-throughput measurement of proteins can be accomplished with technologies such as mass spectrometry. Proteomic studies have found varying protein levels based on brain region. Witzmann and colleagues (2003) describe “subtle but significant” differences in protein expression in rats genetically predisposed to prefer ethanol (i.e., ethanol preferring [P] rats) compared with those bred not to prefer ethanol (i.e., nonpreferring [NP] rats). Indeed, most of the reported protein levels varied only slightly between P and NP rats in both the hippocampus and nucleus accumbens. Potential reasons for the subtlety include limitations in current proteomic techniques such as two-dimensional electrophoresis. In a later study, P rats were allowed ethanol consumption on three different timetables: continuous, at regularly scheduled intervals, or not at all. The researchers reported significant changes in protein levels within the nucleus accumbens and amygdala of differentially treated rats in several molecular pathways, signifying the complexity of effects that ethanol can have on protein expression in varying regions of the brain (Bell 2006).

Though useful, model organism proteomic studies cannot necessarily be directly applied to understanding human systems. Human postmortem proteomic studies have been conducted with samples from the brains of alcoholics. Differences in the proteomes of alcoholics with and without liver cirrhosis were detected in three regions of the brain’s cerebellar vermis, when compared with healthy brains. Thiamine deficiency, which is often related to alcoholism, may be responsible for the changes observed in metabolic protein levels in both groups. Patients with complicated and uncomplicated cirrhosis had differing levels of the structural protein β-actin and the enzymes glutamate dehydrogenase (GDH) and carbonic anhydrase-2, indicating varying effects of liver damage on brain tissue (Alexander-Kaufman et al. 2007).

### Ecology and Environmental Exposure

The genomics era has obvious potential for the study of genetic contributions to psychiatric diseases. Most studies to date have focused on associating polymorphisms with behavior or endophenotypes (Caspi and Moffitt 2006). However, it is well known that environmental influences play a strong role in disease development and are presumed causes of low experimental reproducibility (Hamer 2002). For example, it is anticipated that environmental pathogens such as ethanol modulate the effects of susceptibility genes (Caspi and Moffitt 2006).

A review by Lesch (2005) focuses on serotonin as a link between alcoholism genetics and environment. Based on endophenotypes such as response to ethanol or reaction to stress and anxiety, the authors discuss the role that the serotinergic system plays in modifying each step of the biological hierarchy from genetic basic studies of variants of serotonin to molecular functional imaging. Another review article by Enoch (2006) combines environmental and genetic risk factors into models for high risk of alcoholism. The environmental factors include cultural norms, childhood sexual abuse, and binge drinking as an adolescent. These environmental factors can interact with an individual’s genetic background, making the individual more or less susceptible to genetic risk factors, such as the presence of certain variants of the enzymes monoamine oxidase A (MAOA) or ADH2. Investigating such bridges between gene variants, environment, and endophenotype or phenotype is at the heart of systems genetics and is likely to yield the greatest insight into disease etiology.

Some studies have attempted to investigate the interaction between the genetic and environmental risks for alcoholism. Most studies first try to divide genetic and environmental influences entirely. For instance, a study by Prescott and Kendler (1999) of 3,516 male–male twin pairs revealed that 42 to 52 percent of liability for alcoholism was the result of environmental influences. Finnish twin studies uncovered the gene–environment roles in more detail, suggesting that environment is most important for initiation of drinking, whereas genetic influences are more important for establishing drinking patterns (Rose and Dick 2005). Although it is important that such studies have revealed potential gene–environment interactions, a more thorough understanding of those interactions is required to aid in the development of potential treatment.

Gene–ethanol interaction, though not the only environmental influence potentially involved in addiction, has been most widely studied. Research shows that the risk of becoming dependent increases with increased consumption (Dawson 1994; Whitfield et al. 2004). Most gene–ethanol interactions have been associated with varying response to ethanol consumption with respect to metabolism, intoxication, or enjoyment. Certain ADH and ALDH gene variants are associated with decreased risk for alcoholism. These polymorphisms result in high levels of acetaldehyde (Whitfield 2005), which produces facial flushing and various uncomfortable physiological effects that can reduce the enjoyment of alcohol consumption and therefore reduce risk for dependence.

### Putting the Pieces Together

Systems genetics quickly is becoming a reality because it is possible to measure genetic, genomic, and proteomic factors at different levels of the biological hierarchy between genotype and phenotype. For example, expression quantitative trait locus (eQTL)^9^ mapping attempts to identify QTLs for gene expression (Kendziorski and Wang 2006). In this case, the phenotypes are the expression levels (how actively the genes are undergoing transcription) measured using microarrays. Genetic variants such as SNPs also can be measured in a high-throughput manner and associated with the expression levels of the genes, thereby using genetic variation as a predictor for variation in expression. Researchers particularly are interested in specific DNA regions that are responsible for regulating levels of gene expression (i.e., regulatory regions). *Cis*-acting regulatory regions are located in or near the affected gene, whereas *trans*-acting regulatory regions are located far away and often are master regulators of several genes. Although linkage analysis or expression analysis alone cannot uncover these regions, eQTL mapping with computer programs can narrow the search by combining the two data types and searching for *cis*- and *trans*-acting regulators (Mueller et al. 2006).

Gathering linkage and expression data can be time consuming and the volume of data overwhelming. However, statistical and computation methods are useful for dealing with the data in a streamlined way. Kendziorski and Wang (2006) review available methods and caveats. One tool of note, eQTL Explorer, allows (patho)physiologic QTLs (associated with a pathophysiologic trait) (pQTLs) and eQTLs to be displayed alongside one another, while linking QTL information to outside databases (Mueller et al. 2006). This will enable researchers to link genomic, expression, and physiologic data and understand more fully the interactions that result in alcohol dependence, bridging the gap between genetics and etiology. Although many questions remain as to appropriate statistical handling of data (see Kendziorski and Wang 2006 for a complete review), this mapping technique is becoming an increasingly popular experimental method and has been applied to several complex diseases, including alcoholism. For example, MacLaren and Sikela (2005) analyzed more than 39,000 mRNA molecules (i.e., transcripts) from the cerebellums of Inbred Long-Sleep (ILS) and Inbred Short-Sleep (ISS) mice, which show significant differences in sleep time when given a sedative dose of ethanol. eQTL mapping was used to locate chromosomal regions that could be affecting the transcription of 286 differentially expressed genes. Regions on the ends (i.e., distal) of chromosomes 2 and 4, along with a mid-region on chromosome 7, were determined to be eQTL rich, associated with sleep time, ethanol-induced hypothermia (chromosome 2), and ethanol preference (chromosomes 4 and 7).

### The Role of Model Organisms

Systems genetics approaches to studying the genetic architecture of common human diseases will not be possible without first being applied to model organisms in which the underlying biology is more simple and perturbation experiments are possible. It has been suggested that functional studies of unicellular and other simple organisms are key to learning the “rules” governing epistatic interactions and the development of methods that can accurately detail those interactions (Moore and Williams 2005). Because epistasis has been shown to be a ubiquitous principle of biological systems, simple and more controllable subjects than humans provide an analogous venue of study for epistatic interaction. *Drosophila* has been used for many years to study alterations in ADH and related genes and serves as a highly controllable and alterable system for studying ethanol metabolic genes (Freriksen et al. 1994). The use of *Drosophila* for such studies is controversial, however, because of differences in activity between human and *Drosophila* ADH (Benach et al. 2005).

Studying neurological genes in conjunction with metabolic genes in a way that will be applicable to humans requires a more advanced system. Bennett and colleagues (2006) provide an overview of various mouse models in alcoholism, citing that no mouse model entirely captures the complexity of the disease or known behaviors associated with susceptibility. They instead present a strategy for using several models to dissect the genetics behind various components of the disease, including tolerance, withdrawal, and response to ethanol. The strategy uses QTLs and then maps the genes before applying bioinformatics, haplotype, and expression analysis tools that yield candidate genes. Candidate genes then can be applied to a variety of molecular and biochemical analyses that will further elucidate their function and influence on phenotype, such as creating knockout mice.

Methods investigating systems genetics must be applied to the area of bioinformatics and expression analysis. Large-scale genetic analyses of mice showing alcoholism-like behavior should, in the future, be studied from the viewpoint of complex interactions and should apply methods such as MDR and eQTL mapping, as previously described. Because interactions can be studied in mice in a controllable and defined environment, they will be especially useful in examining how the rules governing interactions change in different environmental contexts. Once mouse studies have characterized some of the governing ideas and identified genes leading to alcoholism, results can be used to guide genome-wide systems genetics studies of alcoholism in humans.

The methods employed in systems genetics can be improved and developed further, especially when relating gene–gene interaction to large-scale proteomic analysis. The current statistical tools used to relate SNP data with protein arrays are highly limited at best. In addition, very few resources are available for the systematic analysis of gene–environment interaction on a large-scale basis. Interdisciplinary and collaborative work will be necessary to drive the development of tools and standards for interpreting their results in an approach that will be relevant to the understanding of alcoholism and lead to medical applications.

## Discussion

The new era of “-omics” approaches, along with the development of bioinformatics tools, has made it possible for researchers to more completely tackle the genetic architecture of common human diseases. Alcoholism is a highly complex disease with many interacting genetic, environmental, and socioeconomic factors. Researchers have long been interested in the biological susceptibilities and protective effects of various genetic sequences on alcoholism. An interactive genome, rather than a stationary one, contributes to the incidence of disease. As researchers begin to implement methods that can analyze interactions and integrate data types, we will be better able to apply research to personalized risk assessment and treatment approaches.

This overview examined certain popular tools and how they can be used to further the understanding of alcoholism. However, one area that requires further methods development is gene–environment interaction. Though epidemiologists have various methods for examining gene–environment interaction, the relative number of studies focusing on applying and evaluating these tools are few.

Various tools are available that take multiple layers of information regarding alcohol-influencing factors into account. Before using these tools, researchers should first focus on finding gene–gene interactions in genome-wide analyses. Once genetic epistasis has been identified, various computational systems genetics methods such as eQTL mapping can be used to integrate other levels of data. Such extensive data gathering and analysis will obviously require collaborative efforts and effective use of preexisting data.

Overall, the current state of systems research offers promising insights into the genetics of alcoholism. With alcoholism’s high heritability and widespread medical and socioeconomic effects, studies resulting in a more complete view of alcoholism and its complexity will be of great value to society and should be a high priority among researchers.

## Footnotes

2 Genomics is the study of the structure and function of an organism’s complete genetic content, or genome.

3 Proteomics is the study of the complete set of proteins produced by an organism (i.e., proteome).

4 Parameters are estimated as part of logistic regression in order to quantify the behavior of independent variables in a statistical model.

5 See glossary, p. 84.

6 The standard error represents the “average” deviation between actual and predicted observations.

7 A naïve Bayes classifier uses probability methods to classify new data points.

8 Phenocopy is the presence of phenotypes in a sample that mimic those caused by genetic influences, but are instead the result of environmental influences.

9 QTL are stretches of DNA that are closely linked to the genes that underlie a particular trait or characteristic.
